# Dynamic scenario of metabolic pathway adaptation in tumors and therapeutic approach

**DOI:** 10.18632/oncoscience.123

**Published:** 2015-02-09

**Authors:** Silvia Peppicelli, Francesca Bianchini, Lido Calorini

**Affiliations:** ^1^ Department of Experimental and Clinical Biomedical Sciences, University of Florence, Istituto Toscano Tumori

**Keywords:** “Warburg effect”, hypoxia-inducible factor-1á, oxidative phosphorylation, proliferation, migration, metabolic-target therapy

## Abstract

Cancer cells need to regulate their metabolic program to fuel several activities, including unlimited proliferation, resistance to cell death, invasion and metastasis. The aim of this work is to revise this complex scenario. Starting from proliferating cancer cells located in well-oxygenated regions, they may express the so-called “Warburg effect” or aerobic glycolysis, meaning that although a plenty of oxygen is available, cancer cells choose glycolysis, the sole pathway that allows a biomass formation and DNA duplication, needed for cell division. Although oxygen does not represent the primary font of energy, diffusion rate reduces oxygen tension and the emerging hypoxia promotes “anaerobic glycolysis” through the hypoxia inducible factor-1α-dependent up-regulation. The acquired hypoxic phenotype is endowed with high resistance to cell death and high migration capacities, although these cells are less proliferating. Cells using aerobic or anaerobic glycolysis survive only in case they extrude acidic metabolites acidifying the extracellular space. Acidosis drives cancer cells from glycolysis to OxPhos, and OxPhos transforms the available alternative substrates into energy used to fuel migration and distant organ colonization.

Thus, metabolic adaptations sustain different energy-requiring ability of cancer cells, but render them responsive to perturbations by anti-metabolic agents, such as inhibitors of glycolysis and/or OxPhos.

## INTRODUCTION

In genetic unstable tumor cells, environmental restrictions may stimulate a rapid emergence of a new panel of different subpopulations that may reach stable proportions until the next selection pressure(s) is encountered. Hence, primary tumors are heterogeneous and contain subpopulations of cells that differ in several abilities, such as sustained proliferative signaling, resistance to cell death, invasion and metastatic dissemination [1]. Proliferation, in particular, occurs irrespective of external stimuli, as genetic and epigenetic alterations favor autocrine growth signaling and pathways supporting nutrient uptake and use. Cancer cell proliferation relays on availability of necessary substrates provided by an adequate blood supply, and, necessarily, space as the two major limiting factors. As the cells accumulate and periods of possible environmental stress may alter rate of proliferation and viability, an additional strategy to survive has to adapt. Thereafter, cancer cells migrate into surrounding tissues following destruction and dissolution of collagen and non-collagenous glycoproteins of extracellular matrix, properties related with high motility and secretion of proteolytic enzymes. Thus, cancer cells have to adjust their energy metabolism to fuel different tasks in which are involved [2-4].

Cancer metabolism is now a hot hallmark in cancer, which needs to be revised in order to clarify causes and consequences [4, 5]. The currently renaissance of cancer metabolism also derives from the observation that many oncogenic signals driving tumorigenesis are key regulators of metabolic network [6, 7]. A classical representation of experimental tumors identifies a well oxygenated tumor cell subpopulation located in the vicinity to blood vessels using lactate to fuel oxidative metabolism and sparing glucose, and a distant hypoxic cells using glucose and producing lactate for a metabolic symbiosis model [8] Herein, we intend to revise and offer an integrated model of metabolic adaptation of cancer cells from proliferation to invasion. This view offers the possibility to disclose new strategies based on the anti-metabolic supporting therapy of tumors.

### Metabolic scenario in tumors (see Fig. 1)

**Fig.1:**
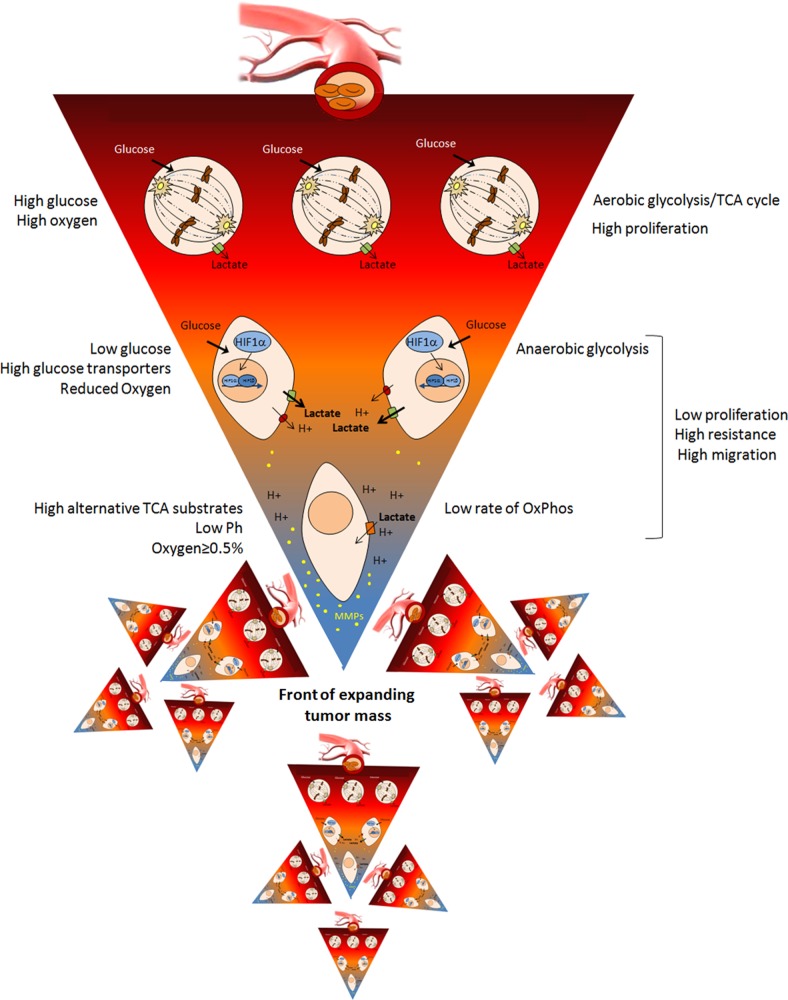
“Inverse pyramid” of metabolic adaptations of tumor cells

Cancer cells undergoing proliferation have to stimulate biomass formation and replicate their genome before to divide into two daughter cells, for that reason the metabolic program of proliferating cancer cells has to satisfy the anabolic demands of macromolecular biosynthesis. Starting from glucose, the glycolytic flux allows generation ATP and biosynthetic precursors of several macromolecules, including those generating lipids, proteins and nucleic acids, necessary to proliferate. This is done via: a) the pentose phosphate pathway and involves the nicotinamtide adenine dinucleotide phosphate (NADPH) generation, used for ribose-5-phosphate and fatty acid synthesis; b) 3-phosphoglycerate (3-PG), which may be used for amino acid and lipid synthesis; and c) the fraction of pyruvate not converted into lactate which furnishes an additional substrate for amino acid synthesis (serine, glycine) and, whether transferred into the tricarboxylic acid (TCA) cycle, generates citrate for lipid biosynthesis and protein acetylation [2, 3, 9]. Therefore, mitochondria participate actively in sustaining biosynthetic pathways in proliferating cancer cells, and an improved uptake of glutamine and glutaminolysis reconstitute TCA intermediates to prevent loss of mitochondrial integrity [10-11].

Overall, a special, but “not” unexpected, characteristic of proliferating cancer cells is a persistence in using specially glycolysis, even in presence of a tension of oxygen able to support oxidative phosphorylation (OxPhos), the so-called “aerobic glycolysis” or “Warburg effect” [9, 12, 13]. It is reasonable whether carbon molecules were entirely oxidized to CO2 in mitochondria, biomass generation from glucose results impossible. Elevated glycolysis in cancer cells represents a likely constitutive phenomenon, independent from oxygen tension. Indeed, c-Myc deregulation is able to promote expression of glucose transporters and glycolytic enzymes [14-16]. The serine threonine kinase Akt is also known as the main inducer of the glucose-lactate pathway, inducing glucose transporter, glycolytic enzymes and phosphorylation and activation of citrate lyase working to increase lipid synthesis [17, 18]. Phosphatidylinositol-3-OH kinase (PI3K), Akt and mammalian target of rapamycin (mTOR) pathway elicit both expression of amino acid transporters and protein translation, thereby coordinating protein synthesis; PTEN acts as a regulator of this flux of information [3, 4, 9]. Furthermore, cancer cells of several tumor histotype express high level and activity of pyruvate kinase (PK) M2 type (PKM2) produced by alternative splicing of the *PKM* gene, which catalyzes the rate-limiting step of glycolysis, controlling conversion of phosphoenolpyruvate (PEP) to pyruvate, and thus ATP generation [6, 19]. By slowing the passage of metabolites through glycolysis, PKM2 promotes the accumulation of large quantities of NADPH and other macromolecules needed to support cell division. PKM2 isoform expression is controlled by c-Myc [14]. Thus, a well-coordinated molecular events may sustain “Warburg effect”.

Glycolysis, although energetically inefficient, relative to mitochondrial OxPhos, can satisfy the energetic yield and pyruvate for TCA cycle of proliferating cancer cells through a more rapid flux and an intense uptake of glucose. It is generally accepted that during tumor progression an inflammatory response operates locally promoting systemic changes, among which a sustained decline in insulin sensitivity would allow the redistribution of glucose from major consumers (e.g. skeletal muscle) to tumor cells. In fact, bioenergetic measurements demonstrate that ATP concentrations in tumors is only slightly modified in respect to normal tissues, and an inefficient ATP generation rises only when substrates are limited [20, 21]. The [^18^F] fluoro-2-deoxyglucose (FDG) positron emission tomography (18F-PET) imaging of tumors reveals glucose uptake by cancer cells. FDG is taken up by glucose transporters and phosphorylated by hexokinase enabling tumor tissue visualization [22]. Besides, imaging technique indicate that around 30% of tumors are not 18F-PET-positive, demonstrating that metabolic profile of tumors is not always glycolytic, and new metabolic tracers are necessary [23].

Although, proliferating cancer cells, which usually located near the vascular tree, use more glucose than oxygen, oxygenation level can be worsened increasing diffusion distance in the tissues. Thus, low oxygen tension areas develop promoting a less proliferating, but more resistant tumor cell phenotype. A key regulator of cellular response to hypoxia is the hypoxia-inducible factor-1 (HIF-1) transcription factor complex, progressively activated in cancer cells by low oxygen tension (mild of 7-21 to severe hypoxia of 1.4-0.7 mmHg) [24]. HIF-1 activity requires the subunit HIF-1α, which works as a master-transcriptional activator for a group of genes involved in cell survival, angiogenesis, migration, energy metabolism and pH regulation [25, 26]. HIF-1α increases migration of tumor cells promoting epithelial- to-mesenchymal transition (EMT) [25], a mesenchymal phenotype expressing invasive and motogenic properties, crucial in local invasiveness and secondary organ colonization [27]. Cells, under HIF-1α control, continue to adopt glycolysis (“anaerobic glycolysis”). HIF-1α renders cancer cells more efficient in mobilizing the residual glucose strengthening glucose transporters (GLUT1 and 3), and stimulates gene transcription of several proteins involved in glucose metabolism, such as aldolase A, enolase-1, esokinase 1 and 3, lactate dehydrogenase A (LDH-A), phosphofructokinase-1 (PFK-1) and PKM2. HIF-1α, concomitantly, represses entry of pyruvate into TCA cycle, inhibiting pyruvate dehydrogenase (PDH) by pyruvate dehydrogenase kinase (PDK) activation [28]. The reduced pyruvate entry into TCA cycle reduces intermediates for biosynthesis necessary for proliferating cells. It has been found that the reduced citrate synthesis can be restored by a significant amount of lipogenic acetyl-CoA provided by glutamine, captured at higher rate by hypoxic than normoxic cells [29]. Thus, HIF-1α may works as a fine-regulator of energy program and migration. HIF-1α is also stabilized by PI3K/Akt/mTOR pathway activation, indicating a converging activation signaling for HIF-1α-dependent glycolysis stabilization [25]. Consequence of these changes is a reduced proliferation, necessary adaptation to survive in a low substrate-containing environment. In fact HIF-1α promotes block of G1/S transition of cell cycle, affecting cyclin-dependent p21 and p27 kinases [30]. HIF-1α has also been demonstrated to increase resistance of cells to apoptosis process [31] and activates Notch and Oct4 signaling that control self renewal and multipotency of stem cells [32]. A risk of anaerobic glycolysis is the increase lactic acid production, that needs to be removed from cells in order to avoid a drop in intracellular pH, that could result in cell damage. To contrast this effect, HIF-1α induces monocarboxylate transporter 4 (MCT4) and carbonic anhydrase IX activity that mediate lactic acid efflux and catalysis of reversible CO_2_ hydration, respectively [26, 33]. Koukourakis and collegue. [34], report that preferential expression of MCT1, which promotes entry of lactate into the cell, and LDH-1 together with elevated PDH activity in tumor fibroblasts support the metabolic use of lactate produced by tumor cells, preventing the development of a hostile acidic environment. The Cori cycle in the liver may also promote reprocessing lactate to glucose, causing a minimum change on energy reserve of whole organism. Nevertheless, in tumors there is a pH gradient with intracellular greater than extracellular pH, reverse of that found in normal tissues. Although acidity is “primarily” caused by endogenous metabolism of cancer cells choosing “Warburg effect” or anaerobic glycolysis, a chaotic vasculature, a reduced intratumoral lymphatic vessels and an altered intratumoral fluid dynamics, due to increased fluid pressure, participate to extracellular tumor acidity development, with a pH value even lower than 6.5 [35].

The reduced pH of tumor microenvironment now triggers in cancer cells, possibly through G-protein-coupled receptors (such as OGR1 and GPR4) [36], signals to stop dividing and gain new vascularized space in adjacent host tissues. Cancer cells with internal pH below 7.1 do not progress into G1/S phase even when an excess of growth factors was applied [37]. Thus, acidic melanoma cells undergo epithelial-to-mesenchymal transition (EMT) [38], just experienced in hypoxic regions, and reprogram their metabolism to a pathway more efficient in ATP production starting from residual energetic substrates. A reduced extracellular pH favors glycolysis inhibition by HIF-1α deregulation [39], and promotes OxPhos pathway [40]. Acidosis inhibits glycolytic enzymes including phosphofructokinase activity, reduces expression of glucose transporters and allows TCA cycle recovering. Unlike cells grown at standard pH (pH 7.3), cancer cells adapted at pH 6.7 do not exhibit “the Crabtree effect” during exposure to high glucose [41]. Low-pH adapted cells exposed to high glucose, instead of to progress toward glycolysis pathway, express a higher pyruvate oxidation [41]. Moreover, it has been demonstrated that glucose depletion leading to a declined ATP production promotes AMP-activated protein kinase (AMPK), a protein kinase complex that regulates cellular energy homeostasis, before cells undergo a state of energetic failure trigging cell death. AMPK activation promotes ATP-generating process such as fatty acid oxidation and electron transport, and inhibits ATP-consuming processes, such as protein, cholesterol and fatty acid synthesis. AMPK-dependent p53 activation negatively regulates cell cycle progression, glycolysis flux through inhibition PI3K/Akt/mTOR pathway, resulting in a p53-dependent metabolic shift toward OxPhos [42, 23]. It has been shown that acidosis through a p53-dependent pathway disconnects ribose synthesis from oxidative pentose phosphate pathway and stimulates glutaminolysis, in order to increase bioenergetics capacity and reactive oxygen species neutralization [43]. It is fascinating to believe that acidosis might play a role in protection of tissues from an ischemic damage. Not yet completely understood is the role played by NF-kB on tumor cell metabolism, although NF-kB is crucial for VEGF-C expression in acidic melanoma cells [44]. OxPhos, whether oxygen tension is still enough (≥ 3.5 mmHg), may work using alternative substrates, such as lactate, free fatty acids and amino acids, to satisfy the high energetic demand of high motile cancer cells. Thus, lactate produced by glycolytic cancer cells, either using Warburg effect or anaerobic glycolysis, constitutes an alternative metabolic fuel for acidic cancer cells expressing an oxidative metabolism. Tumor-associated fibroblasts have been documented to participate in lactate homeostasis in tumors. Fibroblasts in contact with epithelial cancer cells undergo myofibroblast differentiation and produce lactate through aerobic glycolysis which is used by cancer cells for respiration [45-47]. A chronic exposure to low pH (pH 6.5 medium for 8 weeks) stimulates in tumor cells of cervix, colon and pharynx tumors a mitochondrial respiration fueled by glutamine through Sirt1/HIF2α axis [48].

Migration, on the other hand, is a complex process requiring a coordinated action of multiple active macromolecules, implicated in adhesiveness, cytoskeletal dynamic and digestion of extracellular matrix. It was found that melanoma cells reach a maximum of migration at pH 7.0 [49] and acidosis promotes in cancer cells an aggressive phenotype characterized by high resistance to apoptosis, high invasiveness and metastasis, favoring lodgment into target organs [38]. Low extracellular pH favors activation of several proteases, such as metalloproteases (MMP), including MMP-3 and MMP-9, urokinase-type plasminogen activator, cathepsin B and L [35]. MMP-3 and 9 are tightly correlated with the induction of EMT in cancer cells, the characteristic profile adopted by cancer cells to freely migrate into surrounding tissues [38]. Very recently, LeBleu and collegue. indicate that circulating cancer cells undergoing EMT characterized by a high migratory ability and metastatic dissemination, are energetically fuel by mitochondrial biogenesis and OxPhos [50].

On the whole, front of a tumor may expand although at a different rate due to various level of energy obtained from OxPhos activity or HIF-1α-dependent anaerobic glycolysis. Otherwise, when oxygen is not able to sustain OxPhos and environment is anoxic and acidic, cells progress irreversibly to death, justifying the frequent observation of necrosis in spontaneous tumor mass. Whether, cancer cells metastasize distant organs, possibly using OxPhos [50], either they may be dormant or, in case of adequate blood supply, they may reprogram their metabolic profile switching back to an aerobic glycolysis pathway, in order to be proliferative; a profile probably associated with a mesenchymal-to-epithelial transition program. Then, cells again may change to anaerobic glycolysis under the HIF-1α-dependence or acid-stimulated OxPhos to invade locally and gain new space for proliferation.

### Metabolic therapy

Whether tumor metabolism might be considered “the Achilles' Heel of tumors” is yet difficult to say, however metabolic changes, directly or indirectly, accompanied any other property tumor cells acquire during progression to malignancy, such as unlimited proliferation and invasiveness.

Glycolytic inhibitors are not specific and inhibition of glycolysis does not kill tumor cells expressing a different metabolic profile. Among glycolytic inhibitors, is the non-metabolically competitive inhibitor of glucose, the 2-deoxyglucose (2-DG) which is able to suppress hexokinase II and cause a depletion of cellular ATP, leading to blockage cell cycle progression and cell death [51]. However, a dose-dependent toxicity limits its use in human being, although combining 2-DG with adryamicin or paclitaxel, it was found to yield an additive toxicity in human osteosarcoma and non-small cell lung cancers [52]. Other glycolytic inhibitors are: Lonidamine, an exokinase inhibitor; some arsenate compounds, that abolish ATP generation causing arsenolysis in the glyceraldehyde-3-phosphate dehydrogenase reaction; and the 3-bromopyruvate (3-BrPA), another potent inhibitor of hexokinase II, effective in killing liver cancer cells [53]. The ability of 3-BrPA to preferentially kill cancer cells with mitochondrial defects and that live in a hypoxic environment provides a biochemical basis to further develop this class of compounds as novel anticancer agents with potentially promising therapeutic activity and selectivity [54].

MCTs are overexpressed in many tumors and contribute to the maintenance of cellular alkalinity through active export (type 4) or import (type 1) of lactate, pyruvate and ketone bodies. MCT inhibitors have been shown to decrease pHi in neuroblastoma [55] and melanoma cells [56], resulting in cell death. By inhibiting MCT1, lactate is not available to aerobic cells, so it induces a switch from lactate-fuelled respiration to glycolysis, accompanied by a retardation of tumor growth in a mouse model of lung carcinoma and in transplanted human colorectal carcinoma [47]. Several MCT inhibitors have been described but none of them is specific, as cyanocinnamate derivatives (e.g. α-cyano-4-hydroxycinnamate or CHC), bioflavonoids (e.g. phloretin and quercetin), anion transport inhibitors (e.g. niflumic acid and 5-nitro-2-(3-phenylpropylamino) benzoate or NPPB), or stilbenedilsulfonates (e.g. 4,4′-dibenzamidostilbene-2,2′-disulfonate or DIDS) [57]. A recent MCT1-specific inhibitor, AR-C117977, was found to possess immunosuppressive properties that prolongs skin graft and heart allograft survival in mice [58]. MCT1 knockdown suppressed metastatic dissemination of osteosarcoma cells with a mechanism related to NF-kB pathway inhibition [59].

Metformin (N?,N?-dimethylbiguanide) is one of most widely prescribed oral hypoglycemic agent used in type 2 diabetes and also in polycystic ovarian syndrome (PCOS), where insulin resistance is a key factor for metabolic disturbance development. Recently Metformin received increased attention, since its use may reduce cancer risk [60, 61] and may improve cancer prognosis, independently of its hypoglycaemic effect [62]. Metformin acts through inhibition of mitochondrial respiration by inhibiting complex I of electron transport chain and hence is an example of mitochondrial metabolic inhibitor blocking oxidative respiration [63], although metformin may also acts decreasing levels of insulin and insulin-related growth factor. This results in increased cellular AMP-to-ATP ratio which, through AMPK activation, promotes GLUT1 expression [64] and PFK-2 phosphorylation stimulating glycolysis [65]. Metformin-side effect is represented by lactic acidosis, although this effect is dose-dependent and dichloroacetate (DCA) may be used to treat this metabolic effect. DCA may work as anticancer agent working as inhibitor of PDK [66]. Metformin is also a potent blocker of cell proliferation by inhibiting mTORC1 complex [67, 68] and induces cell apoptosis through activating JNK/p38 MAPK pathway [69]. In addition, metformin may upregulate several mRNAs belonging to energy metabolism pathway and downregulates c-Myc, IRS-2 and HIF1α messanger RNAs, effective in reducing incidence and mortality in breast cancer-bearing mice [70]. Combined treatment with metformin and chemotherapeutic agents has been studied using *in vivo* models of breast, prostate and lung cancer. One example is the combination of metformin and doxorubicin, that kills both mammary cancer stem cells and non cancer stem cells [71], or the combination of metformin and 2-DG that has a much stronger deleterious effect than either drug in prostate cancer [72]. As an additional point, it was found that metformin inhibits osteosarcoma growth amplifying the effect of cisplatin [73].

## CONCLUSIONS

It is well recognized that tumor microenvironment, due to an altered and not completely organized vascular network together with a reduced intratumoral lymphatic vessels, suffers from ischemic hypoxia which participates to acidosis development. Thus, tumor cells need to adapt their metabolism to regional variation in glucose and oxygen concentration switching from glycolysis to OxPhos, in order to proliferate or expand into surrounding tissues. Thus, metabolic reprogramming renders cancer cells prone to be perturbed by pharmacological agonists or antagonists disclosing new opportunities for therapy. However, the available metabolic agents are very limited, justifying the search for new products targeting metabolic pathway of tumor cells.
